# Stem Cell-Biomaterial Interactions for Tissue Engineering

**DOI:** 10.1155/2015/126710

**Published:** 2015-06-01

**Authors:** Shuangmu Zhuo, Ming Ni, Long Bi, Lei Xia, Junjun Fan, Hugh H. N. Chan

**Affiliations:** ^1^Fujian Provincial Key Laboratory for Photonics Technology and Key Laboratory of Optoelectronic Science and Technology for Medicine, Ministry of Education, Fujian Normal University, Fuzhou 350007, China; ^2^Institute of Bioengineering and Nanotechnology, 31 Biopolis Way, The Nanos, Singapore 138669; ^3^The Fourth Military Medical University, Xi'an 710032, China; ^4^Department of Physiology, Yong Loo Lin School of Medicine, National University of Singapore, Singapore 117597; ^5^Lerner Research Institute, Cleveland, OH 44195, USA

The design of biomaterials and the sourcing for appropriate cells are two integrated aspects of tissue engineering to construct a tissue implant for clinical applications. During the past decades, many innovative biomaterials with desirable biological and mechanical properties have emerged, while stem cells have been shown to be a promising cell source to differentiate into many cell types. However, the testing of these bioartificial tissue constructs in the clinical trials is far from satisfactory. How microenvironments in the biomaterials regulate cellular signaling pathways and functions, how stem cell-derived target cells respond to extracellular cues presented by the biomaterials, and how implanted tissue constructs interact with host tissues remain to be investigated. The information of the interplay between cell and biomaterials would be helpful to guide us in improving our current strategy to refine the tissue constructs for effective tissue repair in regenerative medicine.

In this special issue, we first present a thorough review by M. Ramamoorthi et al. on the efficacy of dental stem cell therapy in bone regeneration in preclinical* in vivo* and* in vitro* studies. They suggest that well-designed randomized animal trials are needed before moving into clinical trials. Then we introduce three types of innovative biomaterials: (1) bionanocomposites based on bacterial cellulose and magnetic nanoparticles (magnetite) for efficient chronic wounds healing reported by B. Galateanu et al.; (2) the elastomeric poly(*ε*-caprolatone urethane) (PCLU) scaffolds using a high internal phase emulsion for bone tissue regeneration developed by S. Changotade et al.; and (3) a novel gelatin-alginate-polyacrylamide 3D interpenetrating network with superior performance in promoting chondrogenesis using human adipose-derived stem cells designed by S. Dinescu et al. Most importantly, our special focus will be given to the insight studies on the cell-biomaterial interactions. C.-H. Wang et al. demonstrate that the migration ability of bone marrow stem cells can be regulated by varying the porous structure of the artificial ligaments and this regulation is related to the RhoA/ROCK signaling pathway. M. Deng et al. demonstrate the endothelial differentiation of human adipose-derived stem cells on the polyglycolic acid/polylactic acid (PGA/PLA) mesh and propose the synergistic effect of 3D environments and biochemical signals such as growth factors on the acquisition of mature characteristic endothelial phenotype. S. Fu et al. have investigated the protective effect of neuropeptide Substance P on bone marrow stromal cells against apoptosis induced by serum deprivation through Wnt signaling. J. Michel et al. have reviewed a wide range of hydroxyapatite containing scaffolds and their interactions with mesenchymal stem cells in* in vitro* and* in vivo* contexts.

